# Epigenetic changes during hematopoietic cell granulocytic differentiation – comparative analysis of primary CD34+ cells, KG1 myeloid cells and mature neutrophils

**DOI:** 10.1186/1471-2121-15-4

**Published:** 2014-01-20

**Authors:** Rūta Navakauskienė, Veronika V Borutinskaitė, Gražina Treigytė, Jūratė Savickienė, Dalius Matuzevičius, Dalius Navakauskas, Karl-Eric Magnusson

**Affiliations:** 1 Department of Molecular Cell Biology, Institute of Biochemistry, Vilnius University, LT-08662 Vilnius, Lithuania; 2 Department of Chemistry and Bioengineering, Faculty of Fundamental Sciences, Vilnius Gediminas Technical University, LT-10223 Vilnius, Lithuania; 3 Department of General Psychology, Faculty of Philosophy, Vilnius University, Universiteto st. 9/1, LT-01513 Vilnius, Lithuania; 4 Electronic Systems Department, Faculty of Electronics, Vilnius Gediminas Technical University, Naugarduko 41-422, LT-03227 Vilnius, Lithuania; 5 Division of Medical Microbiology, Department of Clinical and Experimental Medicine, Linkoping University, SE-581 85 Linkoping, Sweden

**Keywords:** CD34+ cells, Neutrophils, KG1 cells, Histones, Gene methylation/demethylation

## Abstract

**Background:**

Epigenetic regulation is known to affect gene expression, and recent research shows that aberrant DNA methylation patterning and histone modifications may play a role in leukemogenesis. In order to highlight the co-operation of epigenetic mechanisms acting during the latter process it is important to clarify their potential as biomarkers of granulocytic differentiation.

**Results:**

In this study we investigated epigenetic alterations in human hematopoietic cells at a distinct differentiation stages: primary hematopoietic CD34+ cells, KG1 myeloid leukemic cells, whose development is stopped at early stage of differentiation, and mature neutrophils. We focused on the epigenetic status of cell cycle regulating (*p15*, *p16)* and differentiation related (*E-cadherin* and *RARβ)* genes. We found that the methylation level in promoter regions of some of these genes was considerably higher in KG1 cells and lower in CD34+ cells and human neutrophils. As examined and evaluated by computer-assisted methods, histone H3 and H4 modifications, i.e. H3K4Me3, H3K9Ac, H3K9Ac/S10Ph and H4 hyperAc, were similar in CD34+ cells and human mature neutrophils. By contrast, in the KG1 cells, histone H3 and H4 modifications were quite high and increased after induction of granulocytic differentiation with the HDAC inhibitor phenyl butyrate.

**Conclusions:**

We found the methylation status of the examined gene promoters and histone modifications to be characteristically associated with the hematopoietic cell progenitor state, induced to differentiate myeloid KG1 cells and normal blood neutrophils. This could be achieved through epigenetic regulation of *E-cadherin*, *p15*, *p16* and *RARβ* genes expression caused by DNA methylation/demethylation, core and linker histones distribution in stem hematopoietic cells, induced to differentiation KG1 cells and mature human neutrophils, as well as the histone modifications H3K4Me3, H3K9Ac, H3K9Ac/S10Ph and H4 hyperAc in relation to hematopoietic cell differentiation to granulocyte. These findings also suggest them as potentially important biomarkers of hematopoietic cell granulocytic differentiation and could be valuable for leukemia induced differentiation therapy.

## Background

Epigenetic changes are reversible and interfere with many key biological functions, including regulation of gene expression through chromatin remodeling, DNA methylation/demethylation and microRNA. Moreover, many of these changes have been linked to the pathogenesis of human diseases and cancers [1].

Aberrant DNA methylation is frequent in myeloid malignancies, particularly in the myelodysplastic syndrome (MDS) and acute myelogenous leukemia (AML). Promoter CpG methylation correlates with silencing of tumor-suppressor genes in specific pathways, which are also the targets for mutations or other mechanisms of inactivation [2]. Epigenetic contributions to myeloid pathogenesis appear more complex and deregulations occur at multiple disease stages. Accordingly, therapeutics directed towards epigenetic mechanisms, involving for instance DNA methyltransferase (DNMT) and histone deacetylase (HDAC) inhibitors, have had some clinical success when applied to MDS and AML [2-6].

DNA methylation and histone tail modifications are characteristic epigenetic signatures in physiologic development that become abnormal in neoplasia. Thus, silencing of critical genes by DNA methylation or histone deacetylation can contribute to leukemogenesis as an alternative to deletion or loss-of-function mutations. In AML, aberrant DNA methylation has been observed in several of functionally relevant genes, such as *p15, p16, p73, E-cadherin, ID4* and *RARβ2*. It was shown for instance by Hopfer and coauthors [7] that associations between aberrant promoter methylation and DNMT expression predict high-risk MDS for all lineages and during erythropoiesis. Moreover, hypermethylation of *p15, p16, p73, survivin, CHK2, RARβ* and *DAPK* genes were associated with elevated DNMT isoform expression.

Abnormal activities of histone tail-modifying enzymes have also been seen in AML, frequently as a direct result of chromosomal translocations. It is now clear that these epigenetic changes play a significant role in development and progression of AML, and thus constitute important targets of therapy [8,9]. Interactions between histone modifications and DNA methylation are less well studied. Although genome-wide studies have suggested that there is a negative correlation between H3K4Me3 and DNA methylation, and a positive one between H3K9Me3 and DNA methylation, insights into the understanding of these connections have just recently advanced [10-12].

Hematopoietic stem cells characteristically display self-renewal and differentiation into mature distinct hematopoietic lineages; defining the latter and understanding of the processes that control their differentiation and self-renewal or cause their malignancies are thus of great interest. Human hematopoietic progenitor CD34+ cells collected from healthy human blood, KG1 cells representing blocked differentiation at an early stage of hematopoietic development, and mature human neutrophils can accordingly be used in epigenomic surveys. CD34+ cells provide a valuable model system where progression from quiescent to cycling to differentiated states can be linked to changes in chromatin rearrangements. Changes in histones H3 and H4 modifications being associated with chromatin activation, i.e. H3K4Me3, H3K9Ac, H3K9Ac/S10Ph and H4 hyperAc, and reactivation of methylation-silenced genes could be distinct in hematopoietic primary CD34+ cells, KG1 cells and mature neutrophils. We employed computational analyses of confocal images to evaluate such histone modifications changes in these cell populations.

We disclosed that the rates of methylation in promoter regions of genes involved in the control of differentiation (*E*-*cadherin*, *RARβ*) and cell cycle progression (*p15* and *p16)* were considerably lower than that of unmethylation in CD34+, neutrophils and KG1 cells. As evaluated by computer-assisted methods the H3 and H4 modifications H3K4Me3, H3K9Ac, H3K9Ac/S10Ph and H4 hyperAc were similar for CD34+ cells and human mature neutrophils. The KG1 cells displayed elevated levels of those modifications with an increase after treatment with HDAC inhibitors (HDACI). To conclude, our findings could be important for identification and evaluation of new biomarkers and targets for leukemia differentiation therapy.

## Results and discussion

### Methylation of p15, p16, E-cadherin, and RARβ genes in hematopoietic cells during granulocytic differentiation

Here we chose to examine the methylation status in specific promoter regions of genes involved in cell cycle regulation (*p15, p16*) and granulocytic differentiation (*E-cadherin* and *RARβ*) during hematopoietic cell development. As distinct cellular models we employed human hematopoietic progenitor CD34+ cells collected from healthy human blood, the human myeloid leukemia cell line KG1, whose development is stopped at early stage of differentiation, and mature human neutrophils.

**Figure 1:**
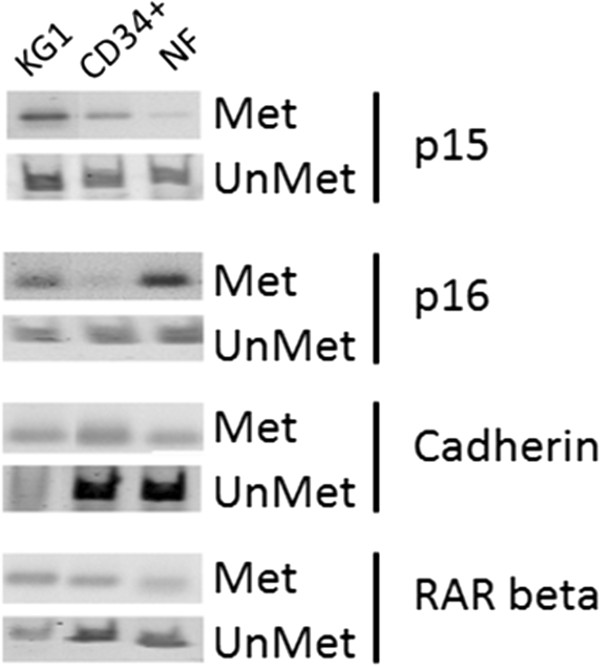
***p15, p16, E-cadherin *and *RARβ *gene methylation status during hematopoietic cell development.** The methylation/unmethylation status was evaluated through genomic DNA bisulfite conversion of examined gene promoter regions, as described in “Materials and Methods”. The products of methylated (Met) and unmethylated (UnMet) *p15*, *p16*, *E-cadherin* and *RARβ* were electrophoresed on 3% agarose gel, stained with ethidium bromide, and photographed. Representative images from one of three experiments showing similar results are shown.

As presented in Figure 1, the hematopoietic progenitor CD34+ cells and mature neutrophils (NF) presented similar demethylation levels of both cell cycle- and differentiation- regulating genes. However, there were lower *p15, E-cadherin, RAR beta* and higher *p16* methylations in human neutrophils than in hematopoietic progenitor CD34+ cells. The promoters of all genes investigated were methylated in KG1 cells. Incidentally, it is known that the INK4 family of proteins p14, p15 and p16 function as cell cycle inhibitors by being involved in the inhibition of G1 phase progression. Methylation of the *p15* promoter is a major gene silencing mechanism in hematological malignancies, while *p14* and *p16* promoter methylations often occur in solid tumors, as well as in leukemia and lymphoma [13,14]. Mizuno and coworkers [15] demonstrated that DNMT genes were constitutively expressed, although at different levels, in T lymphocytes, monocytes, neutrophils, and normal bone marrow cells. Altered expression of DNMT in hematopoietic cells could cause an aberrant methylation/demethylation status of genes in these cells. Using methylation-specific PCR, it was observed that the *p15* gene was methylated in 24 of 33 (72%) cases of patients with AML. Recently we have also shown, that the DNMT inhibitor (DNMTI) zebularine alone or in sequential combination with retinoic acid (RA) decreased expression of DNMT1 in KG1 and NB4 cells, caused partial demethylation of *E-cadherin* and reexpression of pan-cadherin but not the tumor suppressor p15 [16]. We have also demonstrated [17] that DNMTI RG108 changed *E-cadherin* promoter methylation status and the levels of the transcript and protein in NB4 cells. When promyelocytic leukemia cells were treated with RG108 and sodium-4-phenylbutyrate (PB) as single agents and in combination with RA we found [17-19] that these treatments cause increased levels of histone H4 acetylation and methylation of histone H3K4Me3. Both modifications represent an active chromatin state that leads to opening of chromatin structure and induces granulocytic differentiation of human promyelocytic leukemia cells.

Here, we observed that specific promoter regions of genes involved in granulocytic differentiation (*E-cadherin* and *RARβ*) are highly unmethylated both in hematopoietic progenitor CD34+ cells and mature neutrophils (Figure 1). It is known that *E-cadherin* is functionally involved in the differentiation process of cells along the erythroid lineage [20], in CD34+ stem cells and in bone marrow stroma cell [21], and plays a crucial role in cell-cell aggregation during development and could promote intercellular interactions during hematopoiesis. In neutrophils certain promoter regions of *E-cadherin* are highly unmethylated (Figure 1), which relates to differentiation stage of hematopoietic cells.

Corn and others [22] have shown that *E-cadherin* was aberrantly methylated in 4 of 4 (100%) leukemia cell lines, in 14 of 44 (32%) acute myelogeneous leukemias, and in 18 of 33 (53%) acute lymphoblastic leukemias. Methylation was associated with loss of specific *E-cadherin* RNA and protein in leukemia cell lines and primary leukemias. Following treatment with different DNMTIs like 5-aza-2′-deoxycytidine [22] or zebularine [16], leukemia cell line expressed both the *E-cadherin* transcript and protein.

*RARβ* is an RA-regulated tumor suppressor gene silenced by aberrant DNA methylation in acute promyelocytic leukemia (APL) and other human malignancies [23,24]. In human leukemia HL-60 and K562 cell lines *RARβ* gene is silenced [25]. Moreover, using the HDACIs and DNMTIs (TSA, VPA and 5-Aza-CdR, respectively) has been shown to restore the expression of silenced *RARβ*[26,27]. In our study we observed that the *RARβ* methylation/unmethylation ratio in KG1 cells was balanced and constitutes around 50%, whereas in human hematopoietic progenitor cells CD34+ and mature neutrophils *RARβ* promoter regions were methylated only to about 25%.

Our results demonstrate that demethylations in specific promoter regions of *p15*, *p16*, *E-cadherin* and *RARβ* are common phenomena in normal hematopoietic cells and corroborate a hypothesis that methylation of these genes occurs in leukemogenesis.

### Distribution of histones, histone variants and modifications during hematopoietic cell granulocytic differentiation toward mature neutrophils

Core histones H2A, H2B, H3 and H4 wrap DNA and affect chromatin condensation levels through both histone and DNA modifications. The chromatin structure plays an essential role in gene regulation during cell development, proliferation, differentiation and apoptosis, and core histones as well as linker histone H1 variants could be important factors for the maintenance of stem cell pluripotency, DNA condensation and gene expression regulation [28-30]. Indeed, some of histone variants, i.e. H3.3, H2A.Z, H2A.X and macro H2A play precise roles in chromatin structure regulation [31].

**Figure 2:**
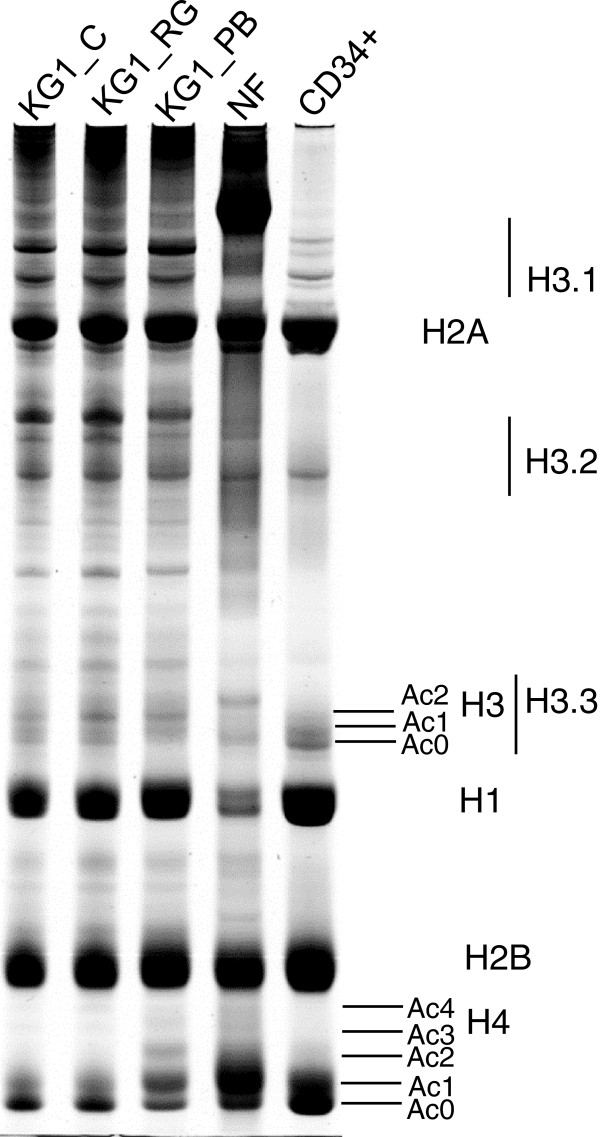
**Histone distribution in hematopoietic cells.** Histones were isolated from untreated (KG1_C), and treated for 24 h with 25 μM RG108 (KG1_RG) or 6 h with 4 mM PB (KG1_PB) KG1 cells, human mature neutrophils (NF) and hematopoietic CD34+ stem cells. Isolated histones were fractionated in an AUT system as described in the section “Materials and Methods”. Representative images from one of three experiments showing similar results are shown.

**Figure 3:**
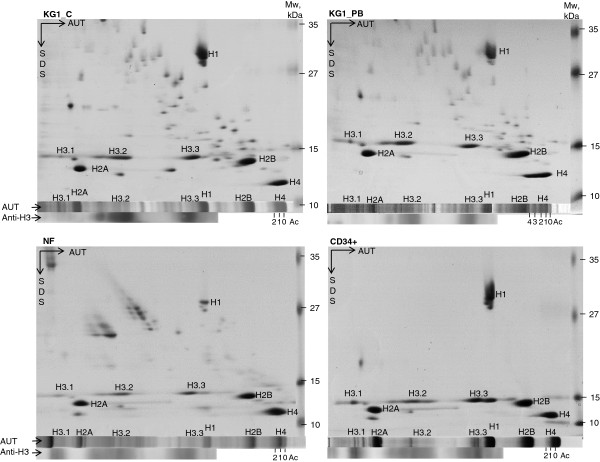
**Histone variants and their modifications during hematopoietic cell development.** Histones were isolated from hematopoietic CD34+ stem cells, myeloid leukemia untreated (KG1_C), and treated with 4 mM PB for 48 h (KG1_PB) KG1 cells, and human mature neutrophils (NF). Isolated histones were fractionated in the AUT system (AUT) as described in the section “Materials and Methods” and then analysed with antibodies against total histone H3 (Anti-H3) or AUT strips were fractionated in SDS/PAGE (SDS). Histones (H1, H2A, H2B, H4), their variants (H3.1, H3.2, H3.3) and histone H4 acetylation forms (Ac 0, 1, 2, 3, 4) are respectively marked in the images. Representative images from one of three experiments showing similar results are shown.

In our study we examined core histone and linker histone H1 distributions in hematopoietic CD34+ stem cells, control and induced to granulocytic differentiation myeloid leukemia KG1 cells and mature human neutrophils. Isolated histones were fractionated in an AUT system and stained (Figure 2) or after fractionation sub-fractionated with SDS/PAGE (Figure 3). In Figure 2 we show that linker histone H1 expression decreased during differentiation and is in a low level in mature human neutrophils. Terme and co-workers [30] have demonstrated that H1 variants are differentially expressed during cell differentiation; here pluripotent cells (ES and iPS4F1) have lower levels of the histone variant H1.0 and higher levels of the H1.3 and H1.5 variants, whereas others, i.e. H1.2 and H1.4, did not display any significant changes.

Histone H3 is an important epigenetic target because of its diverse modification states. In our study we showed that H3.1 and H3.2 are slightly decreased in CD34+ and NF in comparison to KG1 cells represented differentiating hematopoietic cells (Figures 2 and 3). The histone H3 variant H3.3 level did decline only in mature human neutrophils (Figures 2 and 3), where an active gene expression was reduced. It has been shown that H3.3 containing nucleosomes are enriched in active chromatin [32]. Jin and Felsenfeld [33] have demonstrated that H3.3 may play a direct role in activation of the chicken folate receptor (FR) and β-globin genes. As shown in Figures 2 and 3 histones H2A and H2B did not exibit apparent differences in all types of examined cells. By contrast the acetylation of histones H3 and H4 was striking in KG1 cells induced to granulocytic differentiation by HDACI PB and RA [34], but not with DNMTI RG108 (Figure 2).

Our findings suggest that core histones and their variants as well as the linker histone H1 distribute diversely during granulocytic differentiation of hematopoietic cells and that their distribution reflects the differentiation status of hematopoietic cells.

### H3 and H4 modifications highlight active chromatin as being important in hematopoietic differentiation

For evaluation of histone modifications and active chromatin formation during granulocytic differentiation we investigated the modification status of H3K4Me3, H3K9Ac, H3K9Ac/S10Ph and H4 hyperAc histones in human hematopoietic progenitor CD34+ cells, untreated and treated with PB as a HDACI or RG108 as a non-nucleoside DNMTI human myeloid leukemia KG1 cells, and mature human neutrophils.

**Figure 4:**
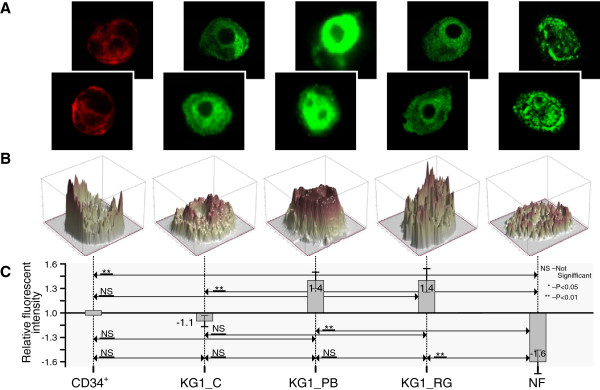
**Histone H3K4Me3 modification levels in hematopoietic cells. A** - Representative confocal images of fluorescence intensity of histone H3K4Me3 in CD34+ cells (CD34+), KG1 control (KG1_C), KG1 cells treated for 48 h with 4 mM PB (KG1_PB) or 25 μM RG108 (KG1_RG) and human mature neutrophils (NF) from two of three experiments showing similar results; **B** - 3D profile of fluorescent intensity of corresponding bottom image from (A); **C** - Ratios of median values of fluorescent intensities are presented. Total fluorescence intensity of each cell category (CD34+, KG1_C, KG1_PB, KG1_RG, NF) was used for computation of median values. Ratios represent fold-change (increased intensity - positive fold-change; decreased intensity - negative fold-change) of KG1_C, KG1_PB, KG1_RG, NF compared to CD34+. The Wilcoxon rank – sum test was used for statistical analysis: *, P < 0.05; **, P < 0.01; NS – no significant change. The bars represent fold enrichment of the modified histones relative to the control (CD34+ cells). Data is the mean ± SD from three independent experiments.

**Figure 5:**
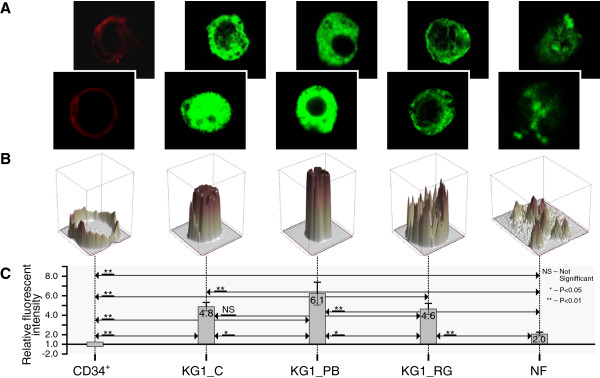
**Histone H3K9Ac modifications during hematopoiesis. A** - Representative confocal images of fluorescence intensity of histone H3K9Ac in CD34+ cells (CD34+), KG1 control (KG1_C), KG1 cells treated for 48 h with 4 mM PB (KG1_PB) or 25 μM RG108 (KG1_RG) and human mature neutrophils (NF) from two of three experiments showing similar results; **B** - 3D profile of fluorescent intensity of corresponding bottom image from (A); **C** - Ratios of median values of fluorescent intensities are presented. Total fluorescence intensity of each cell category (CD34+, KG1_C, KG1_PB, KG1_RG, NF) was used for computation of median values. Ratios represent fold-change (increased intensity - positive fold-change; decreased intensity - negative fold-change) of KG1_C, KG1_PB, KG1_RG, NF compared to CD34+. The Wilcoxon rank – sum test was used for statistical analysis: *, P < 0.05; **, P < 0.01; NS – no significant change. The bars represent fold enrichment of the modified histones relative to the control (CD34+ cells). Data is the mean ± SD from three independent experiments.

**Figure 6:**
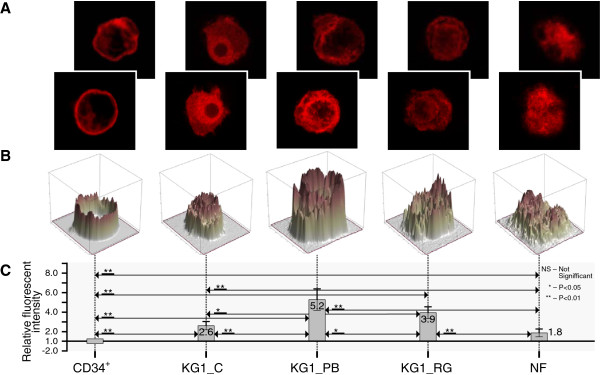
**Histone H3K9Ac/S10Ph modifications during hematopoiesis. A** - Representative confocal images of fluorescence intensity of histone H3K9Ac/S10Ph in CD34+ cells (CD34+), KG1 control (KG1_C), KG1 cells treated for 48 h with 4 mM PB (KG1_PB) or 25 μM RG108 (KG1_RG) and human mature neutrophils (NF) from two of three experiments showing similar results; **B** - 3D profile of fluorescent intensity of corresponding bottom image from (A); **C** - Ratios of median values of fluorescent intensities are presented. Total fluorescence intensity of each cell category (CD34+, KG1_C, KG1_PB, KG1_RG, NF) was used for computation of median values. Ratios represent fold-change (increased intensity - positive fold-change; decreased intensity - negative fold-change) of KG1_C, KG1_PB, KG1_RG, NF compared to CD34+. The Wilcoxon rank – sum test was used for statistical analysis: *, P < 0.05; **, P < 0.01; NS – no significant change. The bars represent fold enrichment of the modified histones relative to the control (CD34+ cells). Data is the mean ± SD from three independent experiments.

**Figure 7:**
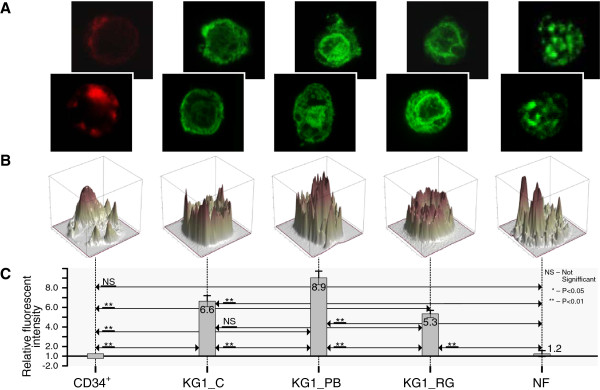
**Histone H4 hyperAc modification during hematopoiesis. A** - Representative confocal images of fluorescence intensity of histone H4 hyperAc in CD34+ cells (CD34+), KG1 control (KG1_C), KG1 cells treated for 48 h with 4 mM PB (KG1_PB) or 25 μM RG108 (KG1_RG) and human mature neutrophils (NF) from two of three experiments showing similar results; **B** - 3D profile of fluorescent intensity of corresponding bottom image from (A); **C** - Ratios of median values of fluorescent intensities are presented. Total fluorescence intensity of each cell category (CD34+, KG1_C, KG1_PB, KG1_RG, NF) was used for computation of median values. Ratios represent fold-change (increased intensity - positive fold-change; decreased intensity - negative fold-change) of KG1_C, KG1_PB, KG1_RG, NF compared to CD34+. Wilcoxon rank sum test was used for statistical analysis: *, P < 0.05; **, P < 0.01; NS – no significant change. The bars represent fold enrichment of the modified histones relative to the control (CD34+ cells). Data is the mean ± SD from three independent experiments.

In Figures 4, 5, 6 and 7 we present confocal images of the fluorescence intensity of histones H3K4Me3 (Figure 4), H3K9Ac (Figure 5), H3K9Ac/S10Ph (Figure 6) and H4 hyperAc (Figure 7) together with ratios of the median values of the fluorescent intensities. Total fluorescence intensity for each cell type grouped by class (CD34+, KG1_C, KG1_PB, KG1_RG, NF) was used for the computation of median values. The hematopoietic progenitor CD34+ cells and neutrophils showed very similar histone modification levels (Figures 5, 6 and 7), except for H3K4Me3; the latter is present in transcriptionally active chromatin and in neutrophils its level was reduced (Figure 4). Moreover, it was diminished in control KG1 cells (Figure 4) in comparison with CD34+ cells. H3K9Me3 deregulation in AML is related preferentially to a decrease of the modifications in core promoter regions. Muller-Tidow and coworkers [35] have shown that a decrease in H3K4Me3 levels at CREs was associated with increased CRE-driven promoter activity *in vivo* in AML blasts. There are also widespread changes of H3K9Me3 levels at gene promoters in AML [35]. Paul and coworkers [36] observed that reactivation of p15INK4b expression in AML cell lines and patient blasts using 5-aza-2′-deoxycytidine (decitabine) and Trichostatin A (TSA) increased H3K4Me3 and maintained H3K27Me3 enrichment at p15INK4b. These data indicate that AML cells with p15INK4b DNA methylation have an altered histone methylation pattern compared to unmethylated samples and that these changes are reversible by epigenetic drugs.

We have demonstrated previously [16] that the DNMTI zebularine induced regional chromatin remodeling by local histone H4 hyperacetylation and histone H3K4 methylation in promoter sites of methylated *E-cadherin* and unmethylated *p21* in promyelocytic leukemia NB4 cells. In this study we also saw increased H3 and H4 acetylated forms both in control and in treated with HDACI and DNMTI KG1 cells. Moreover, PB as a HDACI and RG108 as a DNMTI did not induce KG1 cell differentiation albeit they changed the range of histones H3 and H4 modifications. The elucidation of the epigenetic changes in normal hematopoietic cells and myeloid leukemia cells induced to differentiate will contribute towards the clarification of the histone modifications dynamics in myeloid cell lineage development.

These histone modifications are capable of affecting chromatin structure and gene transcription regulation. Consequently, epigenetic modifiers can be governed in order to regulate repressed genes in leukemia cells. The evaluation of histones H3 and H4 modifications (H3K4Me3, H3K9Ac, H3K9Ac/S10Ph and H4 hyperAc) could be instrumental for finding new leukemia biomarkers on an epigenome basis.

## Conclusions

Evaluation of the methylation status of specific promoter regions of *p15*, *p16*, *E-cadherin* and *RARβ* genes, core and linker histones distribution, histones H3 and H4 modifications (H3K4Me3, H3K9Ac, H3K9Ac/S10Ph and H4 hyperAc) during hematopoietic cell differentiation can provide a new basis for identifying chromatin epigenetic modulators as targets in the regulation of hematopoiesis and for leukemia induced differentiation therapy.

## Methods

### Cell culture

The human myeloid cells KG1 were cultured in RPMI 1640 medium supplemented with 10% fetal bovine serum, 100 U/ml penicillin, and 100 μg/ml streptomycin (Gibco, Grand Island, NY) at 37°C in a humidified 5% CO_2_ atmosphere. In each experiment, logarithmically growing cells were seeded into 5 ml of medium at a density 5 × 10^5^ cells/ml. In the treatment experiments, cells were exposed to the HDACI 4 mM PB or DNMTI 25 μM RG108 the time indicated.

### Separation of mononuclear cells from human blood

Mononuclear cells from whole-blood samples from donors were obtained by buffy coat centrifugation from the blood bank (Linkoping University Hospital, Sweden), see also Ethics Statement. The buffy coat (50 ml) was mixed with 1 vol of 0.9% NaCl and 2 vol of 2% dextran in 0.9% NaCl and allowed the fluid separation for 40 min at 4°C. The upper layer was collected, centrifuged at 300 × g for 10 min at 4°C, the pellet suspended in cold Krebs-Ringer-Glucose (KRG) solution without Ca^2+^ and slowly transferred onto a Lymphoprep (Axis Shield, Oslo, Norway) gradient. After centrifugation at 450 × g for 30 min at 4°C, cells from the mononuclear layer were collected, diluted and washed in cold KRG without Ca^2+^ by centrifugation at 200 × g for 10 min at 4°C. Pelleted erythrocytes were lysed in cold water for 30 sec following a brief addition of 1:3 vol of 3.4% NaCl and 0.55 vol of KRG without Ca^2+^. Mononuclear cells were pelleted, resuspended and washed twice in PBS by centrifugation at 220 × g for 10 min at 4°C.

### Ethics statement

The study was conducted in accordance with the Declaration of Helsinki. Human blood was collected at the blood bank at Linkoping University Hospital by employees at the blood bank division and written consent for research use of donated blood was obtained from all donors. Since blood donation is classified as negligible risk to the donors and since only anonymized samples were delivered to the researchers, the research did not require ethical approval according to paragraph 4 of the Swedish Law (2003: 460; http://www.lagboken.se/dokument/Lagar-och-forordningar/4060/Lag-2003_460-om-etikprovning-av-forskning-som-avser-manniskor?id=64991) on Ethical Conduct in Human Research.

### Isolation of CD34+ cells

CD34+ cells were isolated with the CD34 MicroBead Kit according to the manufacturer’s instructions (Miltenyi Biotec, Germany). Briefly, mononuclear cells were diluted with Isolation buffer containing PBS supplemented with 0.5% BSA and 2 mM EDTA (1:2), and cell clumps were removed by filtering through 30 μM nylon mesh (Miltenyi Biotec, Germany). Then cells were counted and resuspended in Isolation buffer for the up to 10^8^ total cells. Cells were labeled by adding FcR Blocking reagent and CD34 MicroBeads for 30 min at 4–8°C. After centrifugation at 200 × g for 10 min at 4°C, cell suspension was applied onto the LS column, unlabelled cell fraction in the effluent was removed and labeled cells were separated using MidiMACs separator. The purity of isolated CD34+ cells was evaluated by flow cytometry and fluorescence microscopy. Flow cytometric analysis by the use of Becton-Dickinson FACS (Becton-Dickinson FACS Calibur, San Jose, CA) demonstrated a purity of >65% CD34+ cells. For the analysis of histones, the nuclear fraction was isolated from 2.5-3 × 10^6^ CD34+ cells; genomic DNA was prepared from about 4 × 10^7^ CD34+ cells.

### Isolation of neutrophils from healthy human blood

Defibrinated fresh blood was carefully laid on Polymorphprep™ (Nycomed Pharma AS, Oslo, Norway): Lymphoprep gradient (4:1) and centrifuged in swing-out centrifuge at 600 × g for 45 min at room temperature. The uppermost layers down to the granulocyte band were aspirated, and the very diffuse band with granulocytes (neutrophils) collected and diluted with PBS, pH 7.3. After centrifugation at 600 × g for 10 min at room temperature, erythrocytes from the pellet were removed by lysis in water as described above, the pellet of neutrophils resuspended in PBS.

### Histone isolation and analysis

Cells (5 × 10^6^ to 10^7^) were harvested by centrifugation at 500 × g for 6 min, washed twice in ice cold PBS, suspended in Nuclei EZ lysis buffer (Sigma, St. Louis, MO) and nuclei isolated as described by manufacture. For preparation of histones, isolated nuclei were suspended in 5 vol. of 0.4 N H_2_SO_4_ by stirring and incubated overnight at 0°C. The supernatant was collected by centrifugation at 15,000 xg for 10 min at +2°C and the sediment was extracted once more. After centrifugation, both extracts were combined and histones were precipitated by adding 5 vol. of ethanol at -20°C overnight. The precipitated histones were collected by centrifugation, washed several times with ethanol and stored at -20°C until analysis.

Histones (5 μg) were dissolved in a buffer containing 0.9 M acetic acid, 10% glycerol, 6.25 M urea and 5% β-mercaptoethanol, and separated on 15% polyacrylamide gel containing 6 M urea and 0.9 M acetic acid by using 0.9 M acetic acid as a buffer [37]. Histones were detected in AUT system (15% polyacrylamide, 6 M urea, 4 mM Triton X-100 and 0.9 M acetic acid) [38]. After electrophoresis, the gel was stained with Brilliant Blue G-colloidal (Sigma, St. Louis, MO) or blots were probed with primary antibodies against total histone H3 (Abcam, Cambridge PLC.) and secondary antibodies, or fractionated in SDS/PAGE system. Immunoreactive bands were detected by enhanced chemiluminescence according to the manufacturer’s instruction (Western Bright ECL, Advansta Corporation, Menlo Park, CA).

### Bisulfite modification and methylation-specific PCR

The methylation status of gene promoters was determined with the EZ DNA methylation-Direct™ kit (Zymo Research, Irvine, CA). Briefly, cells (1 - 9 × 10^5^) were digested in the reaction mixture with proteinase K at 50°C for 20 min. Bisulfite conversion of DNA was performed according to the manufacturer’s instruction. Thus after conversion of all unmethylated cytosines to uracils, the modified DNA was purified using a Zymo-Spin™ IC column and used for PCR amplification. The primers, forward (F) or reverse (R), for methylated (M) and unmethylated (U) promoters of the target genes were as follows: *E-cadherin* (MF) 5′- CAA TTA GCG GTA CGG GGG GC-3′, *E-cadherin* (MR) 5′-CGA AAA CAA ACG CCG AAT ACG-3′; *E-cadherin* (UF) 5′-TTA GTT AAT TAG TGG TAT GGG GGG TGG- 3′; *E-cadherin* (UR) 5′-ACC AAA CAA AAA CAA ACA CCA AAT ACA-3′; *p15* (MF), 5′-GCG TTC GTA TTT TGC GGT T-3′; *p15* (MR) 5′-CGT ACA ATA ACC GAA CGA CCG A-3′; *p15* (UF), 5′-TGT GAT GTG TTT GTA TTT TGT GGT T-3′; p*15* (UR), 5′-CCA TAC AAT AAC CAA ACA ACC AA-3′. *p16* (MF), 5′-TTA TTA GAG GGT GGG GCG GAT CGC -3′; *p16* (MR) 5′-GAC CCC GAA CCG CGA CCG TAA-3′; *p16* (UF), 5′-TTA TTA GAG GCT GGG GTG GAT TGT-3′; p*16* (UR), 5′-CAA CCC CAA ACC ACA ACC ATA A-3′. *RARβ* (MF), 5′-GGA TTG GGA TGT CGA GAA C-3′; *RARβ* (MR) 5′-TAC AAA AAA CCT TCC GAA TAC G-3′; *RARβ* (UF), 5′-AGG ATT GGG ATG TTG AGA ATG-3′; *RARβ* (UR), 5′-TTA CAA AAA ACC TTC CAA ATA CA-3′. Cycling conditions: 95°C for 5 min, 40 cycles (95°C for 30 s, annealing temperature 66°C (for *E-cadherin* and *p15* Met), 62°C (for *E-cadherin* Unmet and *p16* Met), 57°C (for *p15* and *p16* Unmet, *RARβ*) for 30 s, 72°C for 30 s), 72°C for 10 min. The products were electrophoresed on 3% agarose gel, stained with ethidium bromide, and photographed. The product sizes were as follows: for *p15*, 150 bp; for *E-cadherin*, 170 bp, *p16*, 150 bp, *RARβ* 93 bp. The methylation status of DNA was determined in duplicate samples of three independent experiments.

### Immunofluorescence labeling and confocal laser scanning microscopy (CLSM) of cells

Cover-slips with the captured cells were rinsed three times in phosphate buffer (PBS, pH 7.6) and fixed for 15 min in phosphate buffer supplemented with 3.3% (w/v) paraformaldehyde. Then cells were rinsed three times in PBS, pH 7.6, and permeabilized with 3.3% Triton X-100 for 15 min. The cells were blocked with phosphate buffer containing 5% (v/v) goat serum (DAKO) for 60 min at room temperature. Then, the cover-slips were rinsed and incubated with the indicated primary antibodies against H3K4Me3, H3K9Ac, H3K9Ac/S10Ph (Upstate Biotechnology Inc., Lake Placid, NY) and anti-cd34^+^-FITC (Miltenyi Biotec Inc., Bergisch Gladbach, Germany) for 90 min at 37°C and three times rinsed with PBS, pH 7.6. Finally, the cover-slips that needed were incubated with secondary antibodies, i.e. Alexa 564-coupled goat anti-rabbit or Alexa 488-coupled goat anti-rabbit Fab fragments (Molecular Probes, Eugene, OR) at a concentration 15 μg/ml for visualization.

For confocal imaging, we used a Bio-Rad Radiance 2100 and Radiance 2000MP (Carl Zeiss, Jena, Germany). Images were taken in sequence after inserting the signal enhancing lenses by activating channel 1 (blue); not used: Mai-Tai laser (815 nm), with dichroic beam -splitter 500DCLPXR, blocking filter BGG22 and emission filter D488/10; channel 2 (green): Argon laser (488 nm), no blocking filter and emission filter HQ545/40; and channel 3 (red): Argon laser (488 and 514 nm), no blocking filter and the emission filter E600 LP. The microscope was a Nikon Eclipse TE2000U (Tokyo, Japan), equipped with PlanApo DicH x60 oil immersion objective (NA 1.40).

For visualization of modified histones in CD34+, KG1 and NF three independent biological experiments were carried out. Through observation of the samples around 70-80% of the cells displayed a positive marking of modified histones. Images of representative 4–9 cells from each experiment for each histone modification were taken and summarized in the graphs of the Figures 4, 5, 6 and 7.

### Statistical analysis

Data provided by fluorescence image analysis were not normally distributed, so Wilcoxon rank sum test was used as nonparametric alternative to the two-sample t-test used for independent samples. The Wilcoxon rank – sum test allows a hypothesis test of the equality of two samples medians. *P < 0.05 and **P < 0.01 were considered as statistically significant, and NS describes no significant change. The bar graphs in Figures 4, 5, 6 and 7 represent fold- enrichment of the modified histones relative to the control (CD34+ cells). Data is the mean ± SD from three independent experiments.

### Image analysis

In this research, suit of custom image analysis functions were used. Functions have been implemented in Matlab™ environment (The MathWorks, Natick MA, USA) and were built based on our prototype for 2-Dimensional Electrophoresis gel image analysis [39].

The developed tools were used for fluorescent image analysis, i.e. image preprocessing, segmentation, fluorescent intensity data mining and statistical data analysis. During image preprocessing Gaussian image smoothing is performed for noise reduction. Purpose of segmentation is to acquire spot boundary that delineates cell area from background and other cells. Segmented cell area is used as region of interest (ROI) for spot volume calculations. During segmentation, all available cell layers, that were acquired from microscope, are used. Key tools in segmentation algorithm are symmetrical feature detector and Watershed transformation. Symmetrical feature detector generates map of second order symmetries by the use of the Johansson method [40]. Watershed transformation is used for splitting of symmetry map. After isolation of individual cells, total fluorescent intensity of each cell was obtained.

The protein quantity *V* in a cell is defined as the total fluorescent intensity in a segmented region of corresponding cell. The total intensity of an object is the sum of the intensities of all the pixels that make up the object:

$$V=\sum_{\left(x,y\right)\in \mathrm{cell}}I\left(x,y\right)$$

After quantifying all cells, median values of total fluorescence intensities of each cell grouped by class were computed. The ratios between median values represent fold-change in protein expression. An increase of fluorescent intensity yields a positive fold-change and a decrease, accordingly a negative fold-change. Wilcoxon’s rank sum test was used to evaluate statistically significant changes.

## Abbreviations

AML: Acute myelogenous leukemia; CpG: Cytosine-phosphate-guanine; DNMT: DNA methyltransferase; DNMTI: DNA methyltransferase inhibitor; HDAC: Histone deacetylase; HDACI: Histone deacetylase inhibitor; PB: Sodium-4-phenylbutyrate.

## Competing interests

The authors declare that they have no competing interests.

## Authors’ contributions

RN designed the project, oversaw data generation and wrote the manuscript. VVB carried out the gene methylation/demethylation experiments, performed confocal scanning imaging. DM carried out analysis of confocal images data with computer- assisted methods and data interpretation. GT performed histone isolation, fractionation and analysis. JS isolated CD34+ cells and mature neutrophils from human peripheral blood and prepared KG1 cells for analyses. DN has made substantial contribution to conception and design of image analysis and data acquisition by using computer assisted methods. KEM participated in the experiments with confocal microscopy, the experimental design of the study and revising of the manuscript. All authors read and approved the final manuscript for publication.
